# Developing Preschool Language Surveillance Models - Cumulative and Clustering Patterns of Early Life Factors in the Early Language in Victoria Study Cohort

**DOI:** 10.3389/fped.2022.826817

**Published:** 2022-02-04

**Authors:** Patricia Eadie, Penny Levickis, Cristina McKean, Elizabeth Westrupp, Edith L. Bavin, Robert S. Ware, Bibi Gerner, Sheena Reilly

**Affiliations:** ^1^Melbourne Graduate School of Education, University of Melbourne, Melbourne, VIC, Australia; ^2^Genetics, Murdoch Children's Research Institute, Melbourne, VIC, Australia; ^3^School of Education, Communication and Language Sciences, Newcastle University, Newcastle upon Tyne, United Kingdom; ^4^Deakin University, Centre for Social and Early Emotional Development, School of Psychology, Geelong, VIC, Australia; ^5^Judith Lumley Centre, La Trobe University, Melbourne, VIC, Australia; ^6^School of Psychology and Public Health La Trobe University, Melbourne, VIC, Australia; ^7^Menzies Health Institute Queensland, Griffith University, Gold Coast, QLD, Australia

**Keywords:** language, development, preschool, surveillance, risk factors

## Abstract

**Background:**

Screening and surveillance of development are integral to ensuring effective early identification and intervention strategies for children with vulnerabilities. However, not all developmental skills have reliable screening processes, such as early language ability.

**Method:**

We describe how a set of early life factors used in a large, prospective community cohort from Australia are associated with language abilities across the preschool years, and determine if either an accumulation of risk factors or a clustering of risk factors provide a feasible approach to surveillance of language development in preschool children.

**Results:**

There were 1,208 children with a 7-year language outcome. The accumulation of early life factors increased the likelihood of children having low language skills at 7-years. Over a third of children with typical language skills (36.6%) had ≤ two risks and half of the children with low language (50%) had six or more risks. As the number of factors increases the risk of having low language at 7-years increases, for example, children with six or more risks had 17 times greater risk, compared to those with ≤ two risks. Data collected from 1,910 children at 8- to 12-months were used in the latent class modeling. Four profile classes (or groups) were identified. The largest group was developmentally enabled with a supportive home learning environment (56.2%, *n* = 1,073). The second group was vulnerable, both developmentally and in their home learning environment (31.2%, *n* = 596); the third group was socially disadvantaged with a vulnerable home learning environment (7.4%, *n* = 142); the final group featured maternal mental health problems and vulnerable child socio-emotional adjustment (5.2%, *n* = 99). Compared to developmentally enabled children, the risk of low language at 7-years was greater for children in the three other groups.

**Conclusion:**

The cumulative and cluster risk analyses demonstrate the potential to use developmental surveillance to identify children within the first years of life who are at risk of language difficulties. Importantly, parent-child interaction and the home learning environment emerged as a consistent cluster. We recommend they be adopted as the common focus for early intervention and universal language promotion programs.

## Introduction

Language skills emerge during the first year of life, characterized by periods of swift growth and a relatively consistent sequence of development. Despite these commonalities, a hallmark feature of children's early language development is noticeable individual variability (1). What drives individual child differences in language ability has been a core question of language development research over many decades. Like other developmental domains, language skills are shaped by biological and environmental factors, and while some of the variability in children's language skills can be attributed to both from early in life (2), the ongoing challenge has been to better understand and predict different developmental pathways.

While the earliest years of life provide a crucial window to impact children's developmental pathways (3), building policy for service provision which maximizes learning opportunities in the preschool years requires a comprehensive understanding of a child's development and likely trajectories. Mapping individual developmental pathways involves identification of biological and environmental factors which either serve to buffer a child's development or puts them at risk and in need of preventative or early interventions. Yet ensuring effective early identification processes for some developmental skills has proven elusive. Early language ability is one such example (4) where the current evidence is insufficient to recommend screening in children from birth to 5-years.

Screening and surveillance of development are an integral part of public health approaches which focus on the social and environmental determinants of population health. Public health is defined as “the science of protecting and improving the health of people and their communities” through “detecting, preventing, and responding to disease” (5). The challenge is to ensure the accuracy and reliability of processes for detecting or screening of disease. Understanding the developmental pathways of language ability well enough to inform public health approaches for the management of early language vulnerability and thereby minimize the life-long consequences associated with language disorder, has to date proved impracticable.

This paper draws on almost two decades of data from the Early Language in Victoria Study (ELVS), an Australian longitudinal cohort study, in discussing what we have learnt about predicting language outcomes from early life factors and to question whether novel approaches examining accumulating risks or clustering of risks offer alternative possibilities to the surveillance of preschool language development.

### Developmental Surveillance and Screening

Effective early prevention and intervention efforts have potential for significant positive impacts on children's health and development (6). However, detection processes rely on accurate screening tools to identify those vulnerable children who most stand to benefit from the health and education services available. Currently, not all developmental difficulties have adequately sensitive indicators of vulnerability or measurement tools, and consequently universal screening is not recommended.

Although screening, monitoring and surveillance are terms often used interchangeably, the distinction is critical for complex developmental skills, such as language. Developmental screening involves the use of brief questionnaires and/or standardized tools, used at a designated point in time, to assist in the identification of children who are vulnerable to developmental difficulty. The purpose of developmental screening is to identify children at increased risk of a disorder and who need referral for further in-depth diagnostic assessment (7, 8). Universal screening is recommended when there is a tool sufficiently accurate to ensure confidence in identifying children truly at risk from all children in a certain age group of the population regardless of symptomatology (8), for example, with infant hearing screening. Neither universal nor targeted speech and language screening are currently recommended; two systematic reviews conducted for the US Preventative Services Task Force (4, 9), concluded there was inadequate evidence on the accuracy of screening instruments for speech and language delay. Consequently, research efforts need to consider alternatives to screening, examples of which may include identifying early life factors, and the potential for developmental monitoring (or surveillance) to better detect vulnerable children.

Developmental surveillance refers to a broader, flexible and ongoing process of observing children regularly over time, as well as eliciting and attending to parents' concerns (7, 10). Developmental surveillance, therefore, provides a continuous, collaborative and cumulative process to document developmental history and identify children who may be at risk for developmental problems due to highly individualized and contextual factors. It is the ongoing monitoring of a child's developmental progress which sets surveillance apart from screening (8). While developmental surveillance holds the best opportunity to identify children with vulnerable language skills, there remain significant challenges in what we know, and gaps in how to translate knowledge to primary health and clinical settings.

### Emergence of Language Skills and Language Screening

Language learning occurs within the context of the social interactions and relationships children have with adults and peers in the environment/s in which they live and learn (11–13). Responsive and reciprocal adult-child interactions are critical to the language learning process (14) which best occurs in the context of nurturing, predictable and contingent early experiences with adults (11).

Large scale, longitudinal studies have examined children's developmental pathways and determined a range of factors associated with good vs. poor language outcomes. Over the last 30 years there has been an accumulation of rich language data from international cohort studies, which track the speech and/or language, academic and social-emotional outcomes of participants from early childhood into adolescence and adulthood (15, 16). All of these cohort studies demonstrate that weaknesses in language learning in the preschool and early school years substantially increase the risks of significant later difficulties in education attainment, employment, mental health and wellbeing (17). They also all point to substantial challenges in the precision of early language skills (usually measured by vocabulary at 2-years), to accurately identify those children who will have persistent language difficulties at school entry. Most cohort studies started to document language skills when children were 4-years or older, collecting earlier communication and language milestones retrospectively (18). Many focused on prevalence within clinical samples (19), with few reporting on the association of early life factors with language difficulties (20, 21). These studies consistently found that being male, having a family history of language difficulties, and early neurobiological risks (e.g., low weight for gestational age) were predictors of language status at 2- and 5-years (20, 22).

It was clear that despite the significant work undertaken by the beginning of the twenty-first century, there were still many unanswered questions regarding early life factors, early communication milestones, and emerging language skills at a population level. Equally, early detection was still being considered from a developmental screening perspective, despite emerging concerns regarding the specificity and sensitivity of screening tools (23, 24). While there were a few brief structured screening tools that showed promise (25), many were less accurate at a 2-year follow up or longer (26, 27). There was also the vexed question of whether early detection of language delay actually resulted in short-term health benefits. de Koning et al.'s (28) cluster-randomized trial conducted in the Netherlands concluded that the large-scale introduction of screening for language disorders in toddlers could not be recommended based on both screening and intervention outcomes.

With an understanding of both the findings and gaps from these studies, in 2002 the Early Language in Victoria Study (ELVS) was established to examine the natural history of language development and language difficulties from infancy. Our purpose was to inform policy and practice regarding the promotion of language and communication development, prevention strategies and intervention for young children at risk for language difficulties. In beginning to track children's development in the first year of life in a community representative sample, our aim was to address some of the limitations in the literature at the time.

### The Early Language in Victoria Study

The Early Language in Victoria Study (ELVS), commenced in 2002 with a focus on epidemiology and language skills. ELVS has a prospective, longitudinal cohort design. Our approach was to collect comprehensive information *via* survey from multiple informants that was inclusive of many development domains, as well as through direct assessment of children repeated at several salient ages using “gold standard” measures (29).

ELVS adopted Bronfenbrenner's ecological model to frame and describe the dynamic interactions between child, family, community and broad social-economic and cultural contexts that influence children's learning and development (30). Factors within each of these areas can be either protective, buffering development, or exposing children to risks that can leave them developmentally vulnerable. Consequently, a set of early life risk and protective factors, with proven associations with language outcomes, were derived from the literature and systematic reviews (4, 9) that focused on the child, the family environment, and the primary caregiver (mother).

#### Background

The overall aims of ELVS were to: (i) describe the natural history and clinical course of childhood language disorders; (ii) determine the extent to which language trajectories are fluid, and identify developmental pathways to good vs. low language; (iii) identify which environmental, social and family factors predict variation in these language pathways; and (iv) examine how language pathways are associated with children's social, behavioral and educational outcomes (29). Our objective was to build clinically applicable evidence for the best age at which to accurately identify children who are likely to experience persistent language difficulties. ELVS has followed participants from infancy through to adolescence (13-years). Its current phase is collecting data as participants exit formal schooling (18–19-years). The first phase of ELVS focused on the emergence of language skills up to 4-years, with a second phase extending the research to 7-years.

ELVS analyses draw on data in which language was repeatedly assessed in the preschool and early school years, *via* parent report using the Communication and Symbolic Behavior Scales: Infant-toddler Checklist (CSBS:ITC) (31) and the Macarthur Bates Communicative Development Inventory (CDI) (32) and direct assessment using the Clinical Evaluation of Language Fundamentals (CELF) Australian Adaptations, the Preschool 2nd Edition (33) and Fourth Edition (34). Here, we provide a summary of major themes from the findings when the children were aged between 8-months and 11-years.

#### Most Variation in Language Outcomes Is Unexplained Using Early Life Factors

At 12-months, 2-, 4-, 7, and 11-years (2, 35–38) language outcomes were predicted based on the 12 child, family and maternal factors, and earlier communication and language skills. Our aim was to identify factors that would contribute at key ages to the identification of children with vulnerable language skills. While many of the early life factors remained associated with language outcomes across time, at no point did they provide enough accuracy to identify children with either vulnerable language skills or language difficulties.

A major finding from the 12-month and 2-year analyses was that only a small amount of the total variation in communication and expressive vocabulary scores at each age was explained by the set of 12 risk variables: at 12-months <6.0%, and at 2-years 4.3% (37, 38). Similarly, for vocabulary (number of words produced), the amount of variation contributed by the risk factors was small (7.0% at 2-years). Earlier communication scores made the major contribution to the variance in 2-year vocabulary (38). Notwithstanding this, some of the factors had a significant association with children's communication and vocabulary scores at one or more of the time-points. Table 1 summarizes the significant early life factors at the different ages.

**Table 1:** Summary of significant early risk factors included in regression analyses of language outcomes at ages 2-, 4-, 7-, and 11-years.

**Predictor**	**2-years (CDI)**	**4-years (CELF-P2)**		**7-years (CELF-4)**		**11-years (CELF-4)**	
	**Expressive (*N* = 1,570)^**a**^**	**Receptive (*N* = 1,473)^**a**^**	**Expressive (*N* = 1,442)^**a**^**	**Receptive (*N* = 1,132)^**a**^**	**Expressive (*N* = 1,132)^**a**^**	**Receptive (*N* = 839)^**a**^**	**Expressive (*N* = 839)^**a**^**
	***P***	***P***	***P***	***P***	***P***	***P***	***P***
**Child**							
Male sex	√	√	√	√	√		
Birth weight (per kg)		√	√				
Twin birth					√		√
Preterm birth							
Birth order	√	√	√	√	√	√	√
**Family**							
Non-English- speaking background	√	√	√				
Socioeconomic disadvantage (SEIFA score)		√	√	√	√		
Family history of speech-language difficulties	√	√	√	√	√	√	√
**Mother**							
Maternal education		√	√	√	√	√	√
Maternal mental health							
Maternal vocabulary		√	√	√	√	√	√
Maternal age		√	√				√

*SEIFA, Socio-Economic Indexes for Areas.*

*^a^Children with complete predictor and outcome data*.

More variation in language outcomes was explained by the 12 factors at 4-years than at the earlier ages. For receptive language, the 12 factors together explained 18.9% of the variation, and for expressive language, 20.9%. When we included a measure of Late Talking status at 2-years (based on the 10th percentile cut point for vocabulary), the variance explained increased to 23.6% for receptive language and 30.4% for expressive. Nine of the 12 risk factors were significantly associated with the language scores (see Table 1). Of interest in these 4-year predictive models was the shift from predominantly child factors predicting early communication and vocabulary at 12-months and 2-years, to mainly family and maternal factors significantly predicting language at 4-years. We concluded that the biological (child) factors drive the earliest development (e.g., male sex and birth order), similar to studies that were interested in predicting late talking at 2-years (22). Our 4-year outcomes suggested that the impact of social and environmental factors may take longer to accumulate but are detectable by 4-years. These factors included socio-economic status, family history of language difficulties, and non-English speaking background.

As children progressed through school the same set of potential predictors explained less of the overall variance in language, 9–13% for the receptive and expressive language scores at 7-years and 11–12% at 11-years. Not surprisingly, children's earlier language skills made a greater contribution than the early life factors, low language at 7-years was more accurately predicted by the 4-year language scores, and 11-year language outcomes were predicted more reliably when 7-year language scores were added to regression models, with the variance explained up to 47 and 64%, respectively.

#### Early Variability in Children's Language Profiles

In reporting regularly and systematically on the emergence of children's language skills, findings from ELVS have demonstrated, that despite common assumptions, developmental trajectories fluctuate considerably in the preschool years. This is the case for children with early typical development, as well as for those with early vulnerabilities. In the ELVS cohort, less than half of the children identified at 4-years with language difficulties were identified at 2-years as late talkers (39). These figures are remarkably similar to those from other international cohorts (40, 41). Furthermore, 6% of children who had typical skills at 2-years had language difficulties by 4-years. ELVS analyses also demonstrated that the stability in language classification was low between 4- and 5-years, with 36% of 5-year-olds with low language scores classified as typical at 4-years (42). The variability observed consistently across studies in developmental language pathways to 5-years is the result of a combination of fluctuations in children's abilities, the changing nature of the language skills measured at different ages (e.g., gestures and vocabulary, semantics and grammar), limitations in measurement instruments, and the arbitrary nature of the boundaries defining language difficulties.

#### Developmental Profiles or Sub-groups of Language Trajectories

Latent class modeling has been used to identify sub-groups of developmental trajectories for children within the ELVS cohort using data from 8-months to 4-years and from 4- to 11-years. Developmental profiles were derived from early communication and language measures at 8-months through to direct assessment at 4-years. Five developmental profiles were identified (43): (i) typical group had age-expected language scores at each age (68.5%); (ii) precocious (late) group showed typical development initially but precocity in development from 24-months on (15.0%); (iii) impaired (early) group had high probability of impairment up to 12-months and then typical language development (6.1%); (iv) impaired (late) group, showed early typical development but delay from 2-years (6.1%); and (v) precocious (early) group showed early precocity and typical language by 4-years (4.3%). From these five profiles it was evident that there was considerable variability in the early developmental trajectories in the ELVS cohort. In addition, those profiles in which improvement was shown were more likely to be associated with higher maternal education and vocabulary and less disadvantage, supporting the view that environmental factors have continued impact through the preschool years as language continues to develop. Importantly, the predictors of the developmental profiles pointed to the importance of language enrichment initiatives for more vulnerable children.

Using data from 4- to 11-years, three language trajectory groups were identified (44): (i) a stable group that comprised 94.0% of participants; (ii) a low-decreasing group which included 4.0% of the cohort; and (iii) a low-improving group which included 2.0% of the cohort. The stability in these language trajectories provided confidence for services identifying children with low language at 4-years that they were likely to remain low to 11 years. Further analysis revealed that the low-decreasing group was associated with mainly biological risks, while the low-improving group was associated with mostly environmental risks. In summary, we had demonstrated that by 4-years trajectories stabilize but that prior to that ongoing monitoring rather than discrete screening points was likely to be the best approach to early identification.

#### The Potential of Family and Parent Factors Measured in the First 12-Months

Findings from ELVS across the first 4-years demonstrated that all three sets of early life factors had an impact on language outcomes, but none were as explanatory as vocabulary measured at 2-years, on 4-year language ability. While this finding was not entirely surprising, it did raise the question of whether there was any information collected at 12-months that could have identified children as accurately and earlier than vocabulary at 2-years. A set of risk factors measured at 12-months, derived from the literature and broadly representing child, family and parenting characteristics (45) were used to predict language difficulties at 4-years. The set comprised three child factors: whether the child had started showing objects to adults; the number of words/phrases understood; and the number of words used meaningfully; three family factors: whether there was a family history of speech, language or communication difficulties; maternal education; socio-economic status (SES) quintile; and one factor related to parental communicative behavior. Using these items, measured at 12-months, the model distinguished children with and without language difficulties at 4-years with acceptable discrimination (AUC of 0.73). Whilst by no means diagnostic, this model was substantially better than late talker status at 2-years and had the added advantage of potentially providing an additional 12-month window in which to provide preventative interventions. Given this evidence, we continued to explore these additional family and parental behavior factors.

#### Predictors of Language Growth Over Time

In an effort to better understand the focus and timing of prevention and intervention strategies, ELVS data was used to examine the individual differences in children's language growth over time and identify the factors that best predicted this growth. Twenty-two variables included in the analyses were related to early life factors: child, family and environmental variables; as well as parent reported items from the 4-year parent questionnaire and child assessment, and information collected at other waves (46). The variables were classified into three groups based on whether they were (a) least mutable (not changeable through intervention—for a number of reasons); (b) mutable-distal (could be changed but at a population level through social policy); and (c) mutable-proximal (potential to be modified by direct family or child interventions and with strong evidence that by modifying them a positive impact on children's language can be made). The 22 predictors explained 67.0% of the variability in rate of language growth between 4- and 7-years, with 23.0% contributed by mutable proximal factors, including number of books in the home at 2-years, frequency of shared book reading from 8-months to 4-years, TV viewing at 4-years, and pro-social behavior scores at 4-years. Importantly, not only did the trajectories to 7-years indicate a continued influence of the home environment on children's language development but these mutable proximal factors could be modified through intervention.

#### Summary

After two decades of work with the ELVS cohort we have investigated early life factors, family and environmental characteristics, and expanded factors of interest to parenting communication and behaviors in our efforts to identify those factors that significantly explain later language growth and outcomes at 4- and 7-years. In addition, developmental language profiles to 4-years and language growth trajectories between 4- and 7-years have provided some insights as to who and which factors might be the targets of intervention. It is clear that if developmental surveillance is to successfully identify young children with vulnerable language skills, then we need to identify alternative approaches that consider both cumulative risk and clusters of risk factors. It is our hypothesis based on ELVS data and recent research on developmental vulnerability (47) and language and reading difficulties (48) that developmental surveillance holds the most promise for detecting risk of language difficulties using this cumulative and cluster risk approach.

### Cumulative and Cluster Risk Frameworks Applied to Language Development and Disorder

Any framework to document the most meaningful factors related to language skills needs to be broad and capture those genetic and environmental aspects of the child, their family, and community that impact development. In considering cumulative risk, it is possible to extend our understanding of the complex interactions of biological and environmental factors through the lens of Pennington's multiple deficit model (49). This model has been applied to a range of developmental disorders, including dyslexia (50) and language and reading disorders (49), providing a framework to describe the multifactorial etiologies, while accounting for the accumulation of risk and protective factors determined by multiple and concurrent influences on development. The model assumes that cumulative risk increases the likelihood of emerging developmental difficulties, in a probabilistic rather than deterministic approach. This is compatible with current knowledge on the drivers of language vulnerability.

Furthermore, Shonkoff's bio-developmental framework is complementary and valuable in understanding clustering of risks. This framework is structured into three domains which, across the lifespan, capture the: (a) interactions among foundations of healthy development and sources of early adversity, (b) measures of physiological adaptation and disruption, and (c) both positive and negative outcomes in learning, behavior, and health. Focusing on the “interaction” domain enables us to capture the gene-environment interactions that shape early brain architecture and subsequent outcomes in cognitive, language and social-emotional skills. The interaction domain provides a way of conceptualizing the likely factors that will cluster together to increase language vulnerability and potentially be the levers in successful intervention.

The application of cumulative and cluster risk frameworks has only rarely been used to explain early language vulnerability (48, 51, 52) and few if any studies have had prospective data from the first years of life to determine what factors may be meaningful for developmental surveillance, build a cumulative risk index and determine threshold/s or trigger points at which early intervention is recommended. Moreover, identifying the dynamic and complex interactions early enough that impact language development (i.e., the early life factors which cluster together), can inform the investments and likely targets in interventions for children who are at greatest risk.

Here we want to determine the utility of developmental surveillance, as a way of identifying children at-risk for language difficulties. Using a cumulative risk approach, we wanted to inform the processes of monitoring language development over the first years of life. Moreover, identifying a cluster/s of influential early life factors, we aimed to provide guidance for more customized early interventions.

### The Current Study

The analyses reported here aim to describe how a set of early life factors defined initially in ELVS (9) and added to using a bio-developmental framework (53) are associated with language abilities across the preschool years, and to determine if either an accumulation of risk factors or a clustering of risk factors provide a feasible approach to surveillance of language development in preschool children.

This paper draws on data from ELVS to:

i) Describe the impact of accumulation of a broad set of early life factors up to 7-years of life on language outcomes; that is, does language ability vary based on the number of risks children are exposed to, and does cumulative risk (i.e., an increasing number of factors) improve the accuracy of predicting outcomes?
ii) Investigate clusters of a broad set of early life factors, through latent class analysis, and determine if the latent classes contribute to the accuracy of prediction of language outcomes at 7-years.

## Materials and Methods

The Early Language in Victoria Study (ELVS) commenced in 2002. A community sample of 1,910 infants aged 7.5- to 10-months was recruited between September 2003 and April 2004 from 6 of 31 local government areas (LGAs) in metropolitan Melbourne, Victoria, Australia. The LGAs were selected to represent high, medium, and low SES according to the Australian census-based Socio-Economic Indexes for Areas (SEIFA) Index for Relative Socio-Economic Disadvantage (54).

**Figure 1:**
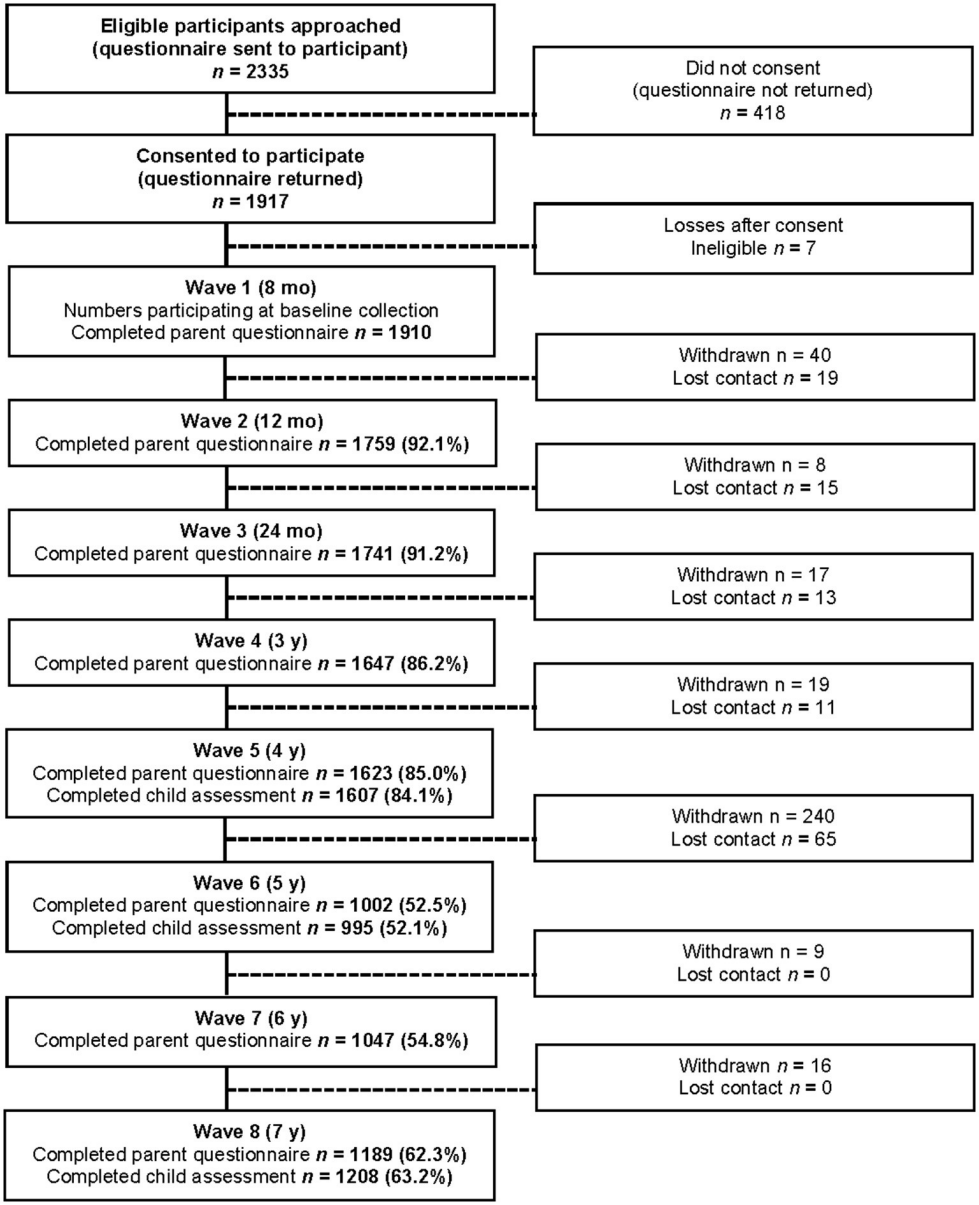
Participant flowchart from baseline (8-months) to 7-years (denominator for percentages is number participating at baseline [*N* = 1,910]). Numbers vary at each wave because of participants' withdrawing, losing contact, or not participating in a particular wave but returning at a later stage.

Infants were recruited through the Maternal and Child Health Service, with supplementary recruitment *via* universally available hearing screening sessions and local newspaper advertising. Infants with developmental delay (e.g., Down syndrome), cerebral palsy, or other serious intellectual or physical disability were excluded, as were parents unable to speak and/or understand English sufficiently to respond to the questionnaires. Further sampling methods and study protocols are reported elsewhere (29). Figure 1 shows participant retention and attrition across the first 8 waves of the study to 7-years. The in-scope sample for this study comprised the 1,208 children from the ELVS cohort who completed direct language assessment at 7-years. This study was approved by the ethics committees of the Royal Children's Hospital (Melbourne) (#23018 and #27078) and La Trobe University (#03-32), and all parents provided written, informed consent.

Parent questionnaire data was collected annually from 1 to 7-years, with parents sent questionnaires within a month of their child's birthday. Face-to-face assessments occurred when children were 4, 5 and 7-years. The face-to-face assessments were administered individually to each child by an experienced trained researcher, usually in a single sitting at the child's local health center, school or home. For these analyses data were drawn from the first eight waves of ELVS questionnaire data and the 4- and 7-year assessments.

### Measures

#### Early Life Factors

A comprehensive set of early life factors included a combination of child (birth weight, non-verbal cognition), family (history of speech/language difficulties, socio-economic disadvantage), maternal (mental health, responsivity) and environmental (home learning environment) characteristics. **Table 3** provides a description of the 16 early life factors and when they were collected. Further details are provided in the following text.

Parents completed study generated questions measuring their child's general health and development, birthweight, family history of speech, language and literacy problems, highest level of parental education, and the main language spoken in the home to the child. Families who reported a main language other than English spoken to the child at home were classified as Non-English-Speaking Background (NESB). Family history of speech and language difficulties was reported at 12-months and coded as positive if the child's father, mother or siblings was reported to have either “been late to talk,” “had ongoing problems with speech or language during childhood,” “had problems with stuttering,” or “had problems learning to read.”

SES was measured using the Australian census-based SEIFA Index of Relative Socio-Economic Disadvantage at the local government area level (mean ± SD of 1,000 ± 100) (54), with lower scores representing greater disadvantage compared with other geographic areas. Maternal mental health was determined by using the Kessler Non-specific Psychological Distress Scale (K-6) (55). Scores were defined as below 4 (“no mental health problem”) and 4 to 24 (“likely mental health problem”). Maternal vocabulary was measured with the written multiple-choice modified version of the Mill Hill Vocabulary Scale (56). Each correct answer is tallied to provide a raw score with a possible maximum of 44, with high scores indicating better vocabulary.

The home learning environment was captured at 2-years by asking parents to report the number of books in the home. Having more than 30 books at home has been found to be an important indicator of child literacy practices at home (57). A study using data from the Longitudinal Study of Australian Children (LSAC) has shown a significant association between the number of children's books available at home and children's reading and numeracy performance (dichotomized variable as 0–30 books and more than 30 books in the home) (58).

Ten items from the Brigance Parent-Child Interactions Scale (BPCIS) (59), shown to predict language outcomes in infants and toddlers, were included in the ELVS parent questionnaire at 12-months, 2- and 3-years. The BPCIS is an 18-item parent-reported measure of parenting behaviors and parents' perceptions about their child, drawn from relevant literature (60). The BPCIS items capture parental responsiveness and responding contingently to a child's needs and interests. The BPCIS total raw scores at each time point were dichotomized to create a high vs. low BPCIS variable. A final categorial variable using the dichotomized BPCIS at 12-months, 2- and 3-years was generated. These groups were categorized as: high responsive parental behaviors consistency score (at or above the median BPCIS total score at three time points); inconsistent responsive parental behaviors score (at or above the median BPCIS total score at one or two time points); and low responsive parental behaviors score (below the median BPCIS total score at three times points).

Child temperament at 12-months was measured using parental ratings on the Approach/Withdrawal scale of the Australian normed Short Infant and Toddler Temperament Questionnaires (61). The Approach/Withdrawal scale produces a total score, with high scores indicating high levels of shyness or low sociability. Social, emotional and behavioral difficulties and prosocial behavior were measured at 4-years using the parent-reported Strengths and Difficulties Questionnaire (SDQ) (62). The SDQ produces a Total Difficulties score (possible range 0–40) and Prosocial Behavior score (possible range 0–10). Prosocial behavior is a protective factor for children with DLD (63), so both scores were included as variables of interest. At 4-years non-verbal IQ was measured by the matrices subtest of the Kaufman Brief Intelligence Test, Second Edition (K-BIT2) (64). The average range for K-BIT2 scores was defined as values not more than 1.25 SDs below the ELVS cohort mean; the internal cutoff point was used because the US normative sample included only 100 children at 4-years. Trained research assistants assessed children in their local child health center or in children's homes.

#### Early Communication and Language Measures

Language abilities were measured using a combination of parent report instruments from 8-months and standardized assessment at 4-and 7-years. At 12-months and 2-years, parents completed the CSBS I-TC (31). This provided a standardized total score (normative mean = 100, SD = 15) and three composite scores for the domains of social, speech, and symbolic skills. The composite domains broadly relate to infants' prelinguistic, linguistic, and cognitive abilities, respectively, each of which has been demonstrated to relate to later expressive language development (65).

Children's gestures were measured at 12-months using the parent-reported CDI Words and Gestures, and included three gesture components: First Communicative Gestures, Games and Routines and Actions with Objects (32). The first two components make up “early gestures,” while the third component is considered “later gestures.” At 2-years vocabulary was measured by the Words and Sentences version of the CDI for infants. Only the expressive vocabulary production percentile was used in this study. Permission was obtained from the authors to substitute 24 vocabulary items to accommodate Australian usage (e.g., “footpath” instead of “sidewalk”).

At 4- and 7-years language was assessed individually by trained research assistants. At 4-years the Australian adaptation of the Clinical Evaluation of Language Fundamentals-Preschool, Second Edition (CELF-P2) (33) was administered and at 7-years children completed the Clinical Evaluation of Language Fundamentals Fourth Edition (CELF-4) Australian Standardization (34). All subtests of the CELF-P2 and 4 were completed. Both the CELF-P2 and CELF-4 composite scores are standardized with a mean of 100, and a standard deviation of 15. Low language outcome was based on a cut point of >1.25 SD below the mean on the CELF-4 Core Language standard score. This cut point has been used in previous ELVS analyses and is in line with other population-based studies in the literature.

### Analysis Plan

#### Cumulative Risk Analysis

For this analysis, cut-off criteria had to be determined for all continuous risk factor variables. As the purpose of dichotomizing the risk factor variables was to describe the impact of cumulative risk factors across the first 7-years of life on language outcomes, we used a more generous cut point of the 20th percentile for continuous variables, rather than clinical cut points. As we wanted to determine whether clusters of risk increase the accuracy of predicting later language ability, we used this cut point to ensure that those children at risk of later language problems would be included. Our approach for managing missing data was a complete case analysis of the data set.

As 16 risk factors were included in our analyses and the proportions of children with some were quite small, we used a grouping strategy reported in Hayiou-Thomas et al. (48) to determine risk categories. This required the smallest risk category was at least as large as the percentage of participants observed with a low language outcome at 7-years (i.e., 10.5%). To examine the association between the cumulative risk categories and low language at 7-years, binomial regression was completed to produce risk ratios (i.e., the risk of low language for children with 3 or more risk factors compared with children with <2 risk factors as the reference group).

#### Latent Class Analysis

We used latent class analysis in Mplus version 8.3 (66) to identify unique subgroups of participants based on a broad set of early life factors. Where possible, we included continuous variables to maximum variation in the model, and otherwise included as binary variables. For consistency, we recoded continuous variables so that high scores equated to higher risk. To identify the optimal number of latent profiles, we began with a two-profile model and added one profile at a time. We selected the optimal number of profiles based on three criteria; first, visual examination of elbow plots of the Akaike Information Criteria (AIC), Bayesian Information Criteria (BIC), and sample-adjusted BIC (SABIC) (67, 68); second, we considered results for the Bootstrapped likelihood ratio test and Lo-Mendell-Rubin Adjusted test (lower *p*-values preferred) (69); and third, we used Nylund et al.'s (70) criterion that the posterior probabilities should be >0.70 as evidence that an individual belongs to their assigned profile and no other. We then ran multiple regression analyses to examine whether the latent classes (with the most advantaged class in regard to early life factors as the reference category) predicted language outcomes at 7-years.

## Results

Of the original ELVS cohort of 1,910 participants, 1,208 (63.2%) completed a direct assessment at 7-years; they are considered the in-scope sample for the analyses reported here. In the cumulative risk analyses, 966 (50.6%) participants had complete data across the 16 early life factors. All 1,910 original ELVS participants were included in the latent class model, however, we predicted risk ratios for the different classes in the model based on the 1,208 who had completed a 7-year language assessment.

The characteristics of the in-scope participant (*n* = 1,208) and non-participant (*n* = 702) groups are presented in Table 2 which illustrates that attrition resulted in a shift in the 7-year cohort characteristics to the original sample. At 7-years, participants were more likely to be English speaking, live in areas of comparatively less disadvantage, and have mothers who were more educated than non-participants and who had higher vocabulary scores.

**Table 2:** Characteristics at baseline of participants and non-participants at 7-years.

**Characteristics^**a**^**	**Non-participants at 7-years** **(*n* = 702)**	**Participants at 7-years** **(*n* = 1,208)**	***P***
**Child**			
Female, %	329 (46.9)	616 (51.0)	0.08
Birth weight (kg), mean ± SD	3.40 (0.6)	3.45 (0.5)	0.04
Twin birth, %	25 (3.6)	28 (2.3)	0.11
Preterm birth, %	20 (2.9)	39 (3.2)	0.64
Birth order, %			0.08
First	340 (48.9)	613 (50.8)	
Second	265 (38.1)	407 (33.7)	
Third	69 (9.9)	157 (13.0)	
Fourth or later	21 (3.0)	30 (2.5)	
**Family**			
Non-English-speaking background, %	80 (11.4)	46 (3.8)	<0.001
Socioeconomic disadvantage (SEIFA score), mean ± SD	1028.02 (67.3)	1040.71 (56.1)	<0.001
Family history of speech-language difficulties, %	181 (25.8)	294 (24.3)	0.48
**Mother**			
Maternal education, % years of completed schooling			
≤ 12 y reference	196 (28.3)	247 (20.7)	<0.001
13 y	285 (41.2)	472 (39.5)	
Degree/postgraduate	211 (30.5)	475 (39.8)	
Maternal mental health, %	178 (30.2)	377 (32.4)	0.35
Maternal vocabulary mean ± SD	26.17 (5.6)	28.19 (4.7)	<0.001
Maternal age mean ± SD	30.94 (4.7)	31.85 (4.4)	<0.001

*P-values were derived through comparisons between those completing 7-year-old assessments and those lost to follow-up by using either χ^2^-tests for categorical variables or t-tests for continuous variables.*

*^a^Baseline represents data collected at 8–10 and 12-months*.

### Cumulative Early Life Factors for Language Development and Difficulties Across the First 7-Years

In the cumulative risk analysis, the proportion of participants presenting with low language abilities was 8.7% (*n* = 84). The CELF core language score of the typical group (*n* = 882) was 100.9, with a standard deviation of 10.1 and a range of 82–134. In contrast, the group with low language had a CELF core score of 72.8 (SD 9.1) and a range from 40 to 81.

Table 3 presents the proportion of the total number of children (*n* = 966) with an individual risk factor, as well as the number of children with only that particular risk factor (e.g., 3.7% of the sample are male but have no other identified risk factor). Other than male sex, the frequency of children with only one individual risk factor was small, ranging from 0 to 1.2%.

**Table 3:** Occurrence of identified risk factors in the overall sample (*N* = 966)^a^.

**Risk factor**	**Age of measure**	**Dichotomized variable**	**Children with this risk factor, *n* (%)**	**Children with only this risk factor, *n* (%)**
**Child**				
Sex	8-months	Males at increased risk compared with females	476 (49.3)	36 (3.7)
Birth weight	8-months	Coded as low birth weight if <2,500 g	39 (4.0)	5 (0.5)
Temperament	12-months	Coded as high shyness/low sociability if in the top 20th percentile	177 (18.3)	6 (0.6)
Emotional and behavioral development	4-years	Coded as emotional and behavioral difficulties if in the top 20th percentile	165 (17.1)	4 (0.4)
Prosocial behavior	4-years	Coded as poor prosocial skills if in the bottom 20th percentile	283 (29.3)	9 (0.9)
Gestures	12-months	Low gestures, bottom 20th percentile	272 (28.2)	7 (0.7)
Non-verbal cognition	4-years	Low non-verbal IQ as standard score, bottom 20th percentile	180 (18.6)	3 (0.3)
Early vocabulary skills	2-years	Coded as low expressive vocabulary if in the bottom 20th percentile	256 (26.5)	10 (1.0)
**Family**				
Non-English- speaking background	4-years	Coded as NESB if main language spoken to child is not English	16 (1.7)	0 (0.0)
Socioeconomic disadvantage (SEIFA score)	8-months	Coded as more disadvantaged if in the bottom 20th percentile	169 (17.5)	2 (0.2)
Family history of speech-language difficulties	8-months	Coded ‘yes' if the child's father, mother or siblings was reported to have either “been late to talk”, “had ongoing problems with speech or language during childhood”, “had problems with stuttering”, or “had problems learning to read”	236 (24.4)	12 (1.2)
Parent-child interaction	12-months, 2-years, 3-years	Coded as low parent responsivity if low on parent-child interaction measure at all three time points	227 (23.5)	4 (0.4)
**Mother**				
Maternal education	8-months	Coded as low maternal education if did not completed year 12	192 (19.9)	9 (0.9)
Maternal mental health	8-months	Coded as “likely to have mental health problem” for those scoring 4 to 24	310 (32.1)	8 (0.8)
Maternal vocabulary	12-months	Coded as low vocabulary if in the bottom 20th percentile	128 (13.3)	4 (0.4)
Number of books in the home (home learning environment)	2-years	Coded as negative if parent reported 0–30 children's books in the home	292 (30.2)	12 (1.2)

*^a^Sample size includes no missing data on risk factor variables and outcome data at age 7*.

Factors related to the child (e.g., early communication and vocabulary skills, children's prosocial capacity), family (e.g., number of books in the home, parent-child interaction), and mother (e.g., maternal education and vocabulary) were represented in the factors with the highest proportions. Early risk factors related to the mother (e.g., maternal mental health) and family (e.g., history of speech-language difficulties) were also represented in factors with the highest proportions.

**Figure 2:**
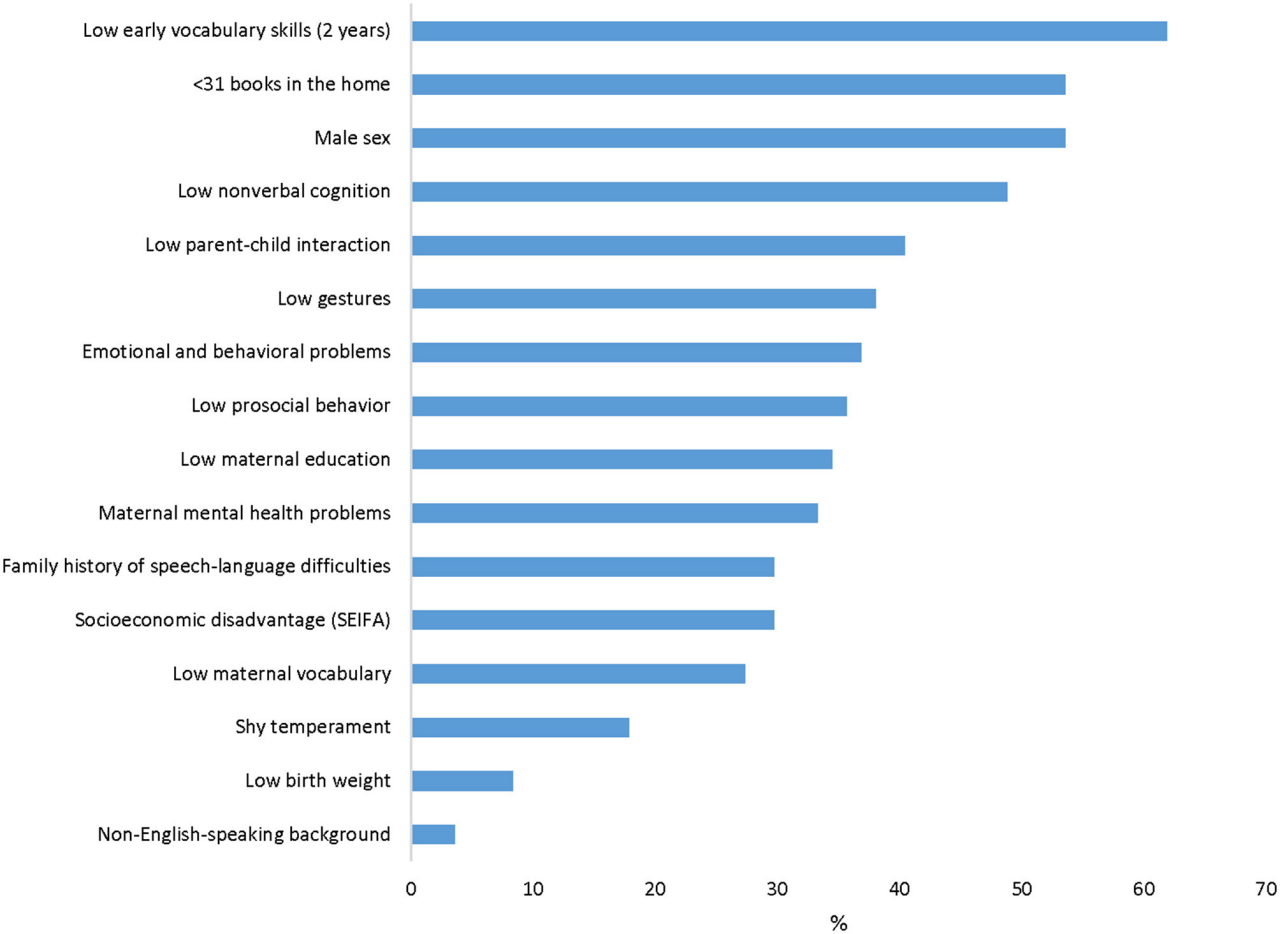
Proportion of children with low language at age 7 by risk factor.

The proportion of children with low language presenting with each risk factor and the ranking of factors is presented in Figure 2. A similar set of factors related to the child (e.g., early communication and vocabulary skills), family (e.g., number of books in the home parent-child interaction), and mother (e.g., maternal education and mental health) were present in more than a third of children with low language.

Data is presented for five cumulative risk categories that were created based on the strategy described earlier (48), with the smallest risk category created representing 14% of the total sample (i.e., five risks). The cumulative risk categories represent the proportion of children with less than or equal to two risks; three; four; five; and six or more risks. Over a third of children with typical language skills (36.6%) were in the ≤ two risks category and half of the children with low language (50%) had six or more risks. Further detail for the other cumulative risk categories is provided in Table 4, along with the risk ratios associated with each category. The results demonstrate that the risk of having low language at 7-years is 17 times more likely for those children with six or more risk factors compared to those with ≤ two risks. As the number of risk factors increased from ≤ two, the risk of having low language at 7-years increased, for example children with four or five risk factors were 5 and 7 times more likely to have low language compared to those with ≤ 2 risk factors.

**Table 4:** Association between number of early life factors and low language outcome at 7-years.

	**Total *N***	**Low language *N* (%)**	**Risk ratio (95% CI), *p***
0-2 risks	328	5 (1.5)	1.00 (reference)
3 risks	180	9 (5.0)	3.28 (1.12–9.64), 0.03
4 risks	162	13 (8.0)	5.26 (1.91–14.51), 0.001
5 risks	135	15 (12.5)	7.29 (2.70–19.66), <0.001
6 or more	161	42 (26.1)	17.11 (6.90–42.42), <0.001
Total N	966	84 (8.7)	

### Latent Class Modeling−4 Groups

**Figure 3:**
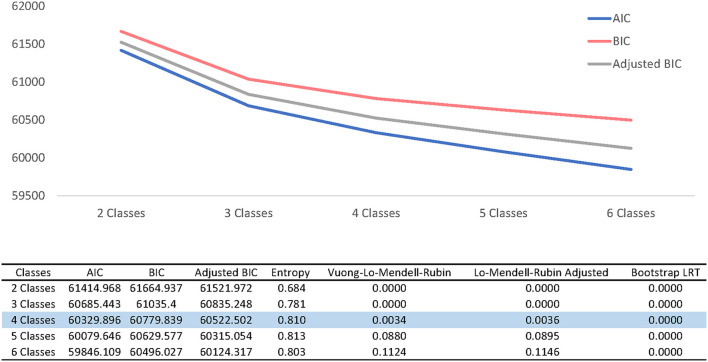
Latent class solutions.

Latent class modeling identified four profile groups. Figure 3 displays the selection process, with information criteria visualized and test results presented for 2–6 profiles, showing an “elbow” at profiles three and four. The four profile-solution was chosen for subsequent analyses because of the large entropy and results from the Bootstrapped likelihood ratio and Lo-Mendell-Rubin Adjusted tests.

**Figure 4:**
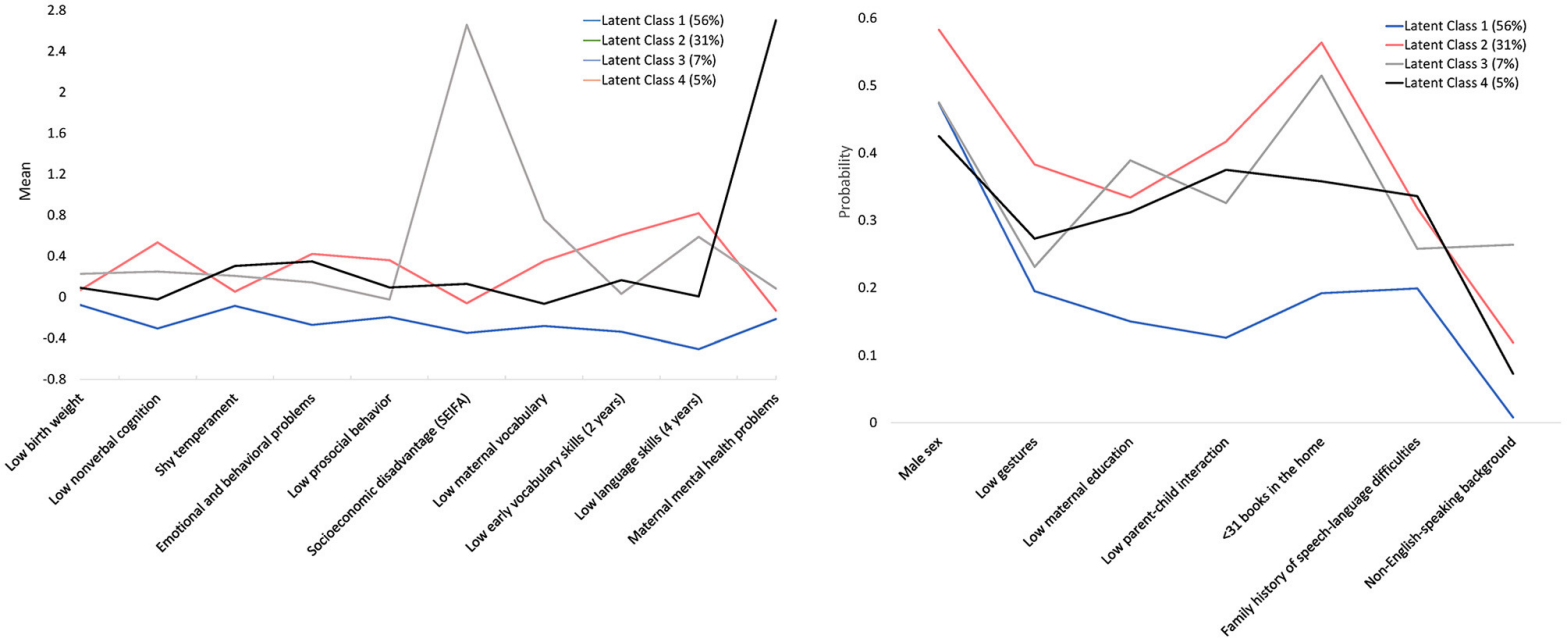
The estimated means (continuous measures) and probabilities (binary measures) for latent classes.

The proportions and mean values of the early life factors for the four classes are displayed in Figure 4, and the distribution of risk factors across the four profiles is shown in Table 5. The first and largest class is developmentally enabled with a supportive home learning environment (56.2%, *n* = 1,073). In comparison to the developmentally enabled group (class 1), the three other groups consistently included more males, low scores for parent-child interactions and low maternal education. In addition, class 2 and 3 both had fewer books in the home and lower language scores at 4-years.

**Table 5:** Distribution of risk factors across the four classes identified using Latent Class Analysis (*N* = 1,910)^a^.

**Risk factor**	**Class 1 (%)**	**Class 2 (%)**	**Class 3 (%)**	**Class 4 (%)**
	**(*N* = 1,073)**	**(*N* = 596)**	**(*N* = 142)**	**(*N* = 99)**
	**56.2%**	**31.2%**	**7.4%**	**5.2%**
Male sex	47.2	58.2	47.9	44.4
Low birth weight	3.3	5.2	7.3	4.1
Shy temperament	20.8	26.9	38.7	29.3
Emotional and behavioral problems	20.6	44.8	43.0	32.3
Low prosocial behavior	19.9	35.1	21.8	32.3
Low gestures	18.6	39.4	23.9	30.3
Low non-verbal cognition	10.2	41.4	27.5	18.4
Low early vocabulary skills at 2-years	16.1	54.0	31.3	36.6
Low language skills at 4-years	1.5	51.3	43.1	18.4
Non-English-speaking background	0.2	6.8	14.9	3.3
Socioeconomic disadvantage (SEIFA score)	8.5	23.7	100.0	26.3
Family history of speech-language difficulties	19.8	32.2	25.4	35.4
Low parent-child interaction	12.2	42.1	33.0	41.1
Low maternal education	14.2	35.4	38.7	30.6
Maternal mental health problems	25.1	30.6	34.7	100.0
Low maternal vocabulary	9.5	29.7	38.7	18.2
Number of books in the home (home learning environment)	18.5	57.9	51.7	36.6

*^a^Sample size ranges from 1,510 to 1,910*.

The second class is described as vulnerable, both developmentally and in their home learning environment (31.2%, *n* = 596); the third class is described as socially disadvantaged, with a vulnerable home learning environment (7.4%, *n* = 142); the final class features maternal mental health problems and vulnerable child socio-emotional adjustment (5.2%, *n* = 99). Each class had a unique and defining feature: for the vulnerable group (class 2) children presented with low use of early gestures, vocabulary and non-verbal cognition; class 3 was set apart from the other groups due to greater socio-economic disadvantage; while class 4 was the only group where more mothers presented with mental health problems.

To investigate whether the latent classes could predict language outcomes at 7-years we conducted binomial regression analyses, including the 1,208 participants with a 7-year language outcome (see Table 6). The proportion of participants at 7-years with low language abilities was 10.5% (*n* = 127). The CELF core language score of the typical group (*n* = 1,081) was 100.6, with a standard deviation of 10.1 and a range of 82–135. In contrast the group with low language had a CELF core score of 71.9 (SD 10) and a range from 40 to 81. Compared to developmentally enabled children (class 1), the risk of low language at 7-years was 13 times greater for children in the developmentally vulnerable group with an insufficient home learning environment (class 2), eight times more likely for those children experiencing social disadvantage, low maternal education and insufficient home learning environment (class 3) and five times more likely for children whose mothers had more mental health problems (class 4).

**Table 6:** Association between the four identified latent classes and risk of low language at age 7-years using binomial regression.

**Latent class**	**CELF Core language score M (SD)**	**Risk ratio (95% CI)**	***p***
Class 4 Maternal mental health problems	98.9 (12.6)	5.90 (2.60–13.40)	<0.001
Class 3 Socially disadvantaged	91.1 (17.9)	8.49 (4.07–17.71)	<0.001
Class 2 Developmentally vulnerable	86.5 (11.4)	13.67 (8.05–23.23)	<0.001
Class 1 Developmentally enabled	107.4 (9.8)	Reference group	

## Discussion

This paper sought to determine the utility of developmental surveillance, as a way of identifying children at-risk of language difficulties. Using a cumulative risk approach, we wanted to inform the processes of monitoring language development over the first years of life. Moreover, identifying a clusters of influential early life factors, we aimed to provide guidance for tailored content for early interventions.

Cumulative risk categories revealed that the risk of low language outcomes at 7-years was significantly increased (from 5 up to 17 times more likely) when risks accumulated, with four, five, and six or more factors present. A broad range of early life factors were represented across all the cumulative categories including characteristics of the child, the family and the environmental context in which the child lives and learns, as well as maternal characteristics and parenting behaviors. These results confirmed one of the assumptions of Pennington's multiple deficit model, that the accumulation of factors increases the probability of developmental difficulties emerging. The findings also align with Hayiou-Thomas et al. (48) who found that cumulative risk was a core component of predicting language and reading difficulties in children at 12-years, using language and family history measures at 4-years. We included a broader set of early life factors, many measured in the first year of life, to investigate the “tipping point” for language vulnerability. From our analyses the accumulation of four or more risks was the critical point where children's risk of later language difficulties was significantly increased.

Several of the most frequently present factors are worth noting. Reflecting the home learning environment, the number of books in the home was consistently important in the current findings and replicates previous work with ELVS (45, 46), LSAC (i.e., frequency of reading to children) (71, 72) and clinical cohorts (73). In a bio-ecological framework this characteristic of parent behavior (i.e., number of books, frequency of reading) represents how the home learning environment can facilitate language learning strategies such as joint attention, labeling, expansion and responsive questioning. It is also the case that capturing this parent behavior during a child's first years is easier to measure than conducting parent-child observations during regular developmental monitoring. Furthermore, maternal resources, such as mental health and education level, influence parent-child interactions, specifically responsivity and reciprocity, features known to be important for language development (14, 74). This relationship has also been identified in studies using qualitative methods of clustering of risks to predict response to an early intervention (75). Early communication skills (e.g., gestures) and vocabulary are also key drivers of later language (2, 38) and need to be included in any subset of factors recommended for developmental surveillance.

All four developmental profiles in the latent class analysis were consistent with the “interaction” domain of the bio-developmental framework, where the foundations of healthy development (genes: “g”) interact with sources of early adversity (environment: “e”). The profiles all represent a combination of the two interaction components, whether advancing outcomes for children with typical development (g) in supportive home learning environments (e) (class 1) or by weakening outcomes for vulnerable children, developmentally (g) and in their home learning environments (e) (class 2), or children who are growing up in disadvantaged circumstances with vulnerable home learning environments (e) where maternal education and language is low (g & e) (class 3). The fourth developmental profile is primarily driven by genetic factors, with vulnerable maternal mental health, child development (g) and low maternal language (g & e) (class 4). Attributing limited maternal resources to both a genetic and environmental source we relied on previous studies reported in the literature. The shared genetic and environmental influence of maternal resources was demonstrated in studies with twins (76). Pathways between parental language input and child language outcomes were determined by both substantial shared genetic influence and “child to parent” and “parent to child” relationship effects.

All four classes with these differing developmental profiles contained children with language scores within one standard deviation of the mean, considered within the typical range. We interpret this finding within a necessary but not sufficient framework, where risks can render development vulnerable but not for all children and not always at clinically diagnostic levels. For example, children growing up in socially disadvantaged circumstances do not all have developmental language difficulties, however, it is a known risk (77, 78). In our cluster model, the greatest variability in language scores was in class 3 (i.e., socially disadvantaged group, with a vulnerable home learning environment). It is important to note that a wide variety of language environments exist in families living with social disadvantage (i.e., not all socially disadvantaged children are exposed to vulnerable home learning environments). Of the classes considered “at risk” the highest mean language score was in class 4 (i.e., children from the maternal mental health and vulnerable child socio-emotional adjustment). We measured maternal mental health at 8-months and our findings suggest that the impact this factor has on language outcomes weakens as children get older. This is consistent with the findings of Taylor et al. (79) who found that maternal mental health distress was associated with higher rates of vocabulary growth between 4- and 8-years. It is possible, that children in this group had more fundamental issues with social-emotional development and adjustment, for which language, was one observable indicator, but not the primary source of vulnerability.

The developmental profiles in this study were comparable with previous work but demonstrated consistent gene-environment interactions unlike work from Christensen et al. (51) who demonstrated qualitatively different clusters of risks associated with vocabulary growth from 4- to 8-years. Six classes were included in the model representative of either environmental only (e.g., “working poor families”), genetic only (e.g., “developmental delay”), or interactions between both (e.g., “overwhelmed”). Similarly, developmental vulnerability at 5-years (47) and reading difficulties in later childhood (52) have been explained by latent class models with 5 and 4 classes, respectively. In both these models there was a mix of genetic only (e.g., child development risk) or environmental only (e.g., socioeconomic risk) classes, as well as classes derived from interactions between gene and environment characteristics (e.g., birth, sociodemographic and health behavior risks). Language skills measured at 7-years are complex including comprehension and expression of vocabulary, grammar, and semantic knowledge and are therefore, qualitatively different from vocabulary only and developmental vulnerability measured in previous studies. The socio-cultural and biological nature of language learning makes it particularly sensitive to both gene and environmental influences which may account for the consistent representation of both factors in the classes identified in our model.

Previous ELVS analyses at 4-, 7-, and 11-years have demonstrated associations between some early life factors and later language outcomes. However, due to large amounts of unexplained variance in language outcomes and the early instability in language profiles, the specific recommendations related to developmental surveillance and intervention were limited and under specified. Analysis of language growth curves from 4- to 7-years was more informative with respect to intervention levers. The inclusion in the present analyses of proximal and modifiable factors important for language outcomes together with early life factors provides compelling evidence to monitor children at-risk for later language difficulties based on a cumulative risk approach. In addition, the developmental profiles (classes) provide information about the content of early prevention and intervention strategies, which our findings suggest need to focus on the home learning environment and parent-child interactions. In the following sections we provide clinical implications and recommendations as they pertain to our findings.

### Implications for Developmental Surveillance (Cumulative Risk)

Developmental surveillance provides the opportunity for flexible and continuous monitoring of development to meet the public health goal of detecting, preventing, and responding to specific disorders in the population. The present analyses suggest that surveillance based on just one to two risk factors will not be helpful in identifying young children with vulnerable language skills. Instead, through observing whether children have four or more accumulated risks, identification will be more accurate given the significant risk ratios associated with language outcomes at 7-years in our cohort. Importantly, most of the factors we considered are easily observed and reported by parents and/or non-specialist health and education staff who know the child. While observations of parent-child interaction are considered the gold standard measure, it is promising that parent report of interaction behaviors measured using the BPCIS identified those parents and children who may benefit from parent-child interaction intervention targeting responsive behaviors.

Specifically, a set of factors that include information about the child's early communication skills, the home learning environment, and parent-child interaction will be well-placed to identify children whose language development may be vulnerable and in need of ongoing monitoring. We recommend universal and regular monitoring throughout the preschool years and beginning in the first year of life, based on our current analyses and previous work (45). Leveraging off well-child health visits it is feasible that a set of factors could be included in a cumulative risk index for language development. While maternal and child health services provide an excellent opportunity to monitor during the very early years, from birth to 2-years, there is often a significant drop-off in families attending routine child health checks beyond this age and early childhood education and care contexts become more important for families and have wider uptake. Consequently, collaboration and seamless transitions from health monitoring to early childhood education services becomes vital to maintain contact with vulnerable children. Policy development that seeks to build collaborative partnerships and sharing of child data between early childhood health and education services is critical here.

### Implications for Targeted Interventions (Latent Class Analysis)

The four developmental profiles represented in the classes identified are all characterized by interaction between genetic and environmental factors. Plomin (80) suggests that “genes are the major systematic force in children's development,” going on to discuss how environmental factors influence development in ways that are not systematic but individual and contextualized; in this view the continuity of development is genetic and change is environmental (81). Consequently, intervention strategies focused on shifting developmental trajectories should first and foremost consider the environmental factors most likely to change and impact language abilities. Earlier ELVS findings and the current latent class model suggest that there are common goals for all children with vulnerable language abilities, specifically related to parent-child interaction and the home learning environment. Further, our findings speak to the need for individualized supports and strategies provided for sub-groups of at-risk children and families. Importantly, it is clear that across the developmental profiles, a key priority for intervention is the nurturing of parent-child relationships and responsive and reciprocal interactions. To achieve this outcome for families with differing developmental profiles will require tailored and personalized approaches to intervention.

Pleasingly, a recent systematic review and meta-analysis has demonstrated that parent-child interaction interventions that focus on promoting responsive parenting demonstrate greater effects on child cognitive development, parenting practices, and parent-child interactions when compared with interventions without a focus on responsive parenting (82). Universal early childhood services, such as maternal and child health, could utilize a targeted intervention approach following developmental surveillance. Our findings demonstrate that both child language and parent-child interaction measured through parent report can detect vulnerabilities and indicate the need for responsive parenting interventions. Observational tools, such as the Parental Responsiveness Rating Scale, provide an efficient, reliable method for practitioners to measure parental responsiveness during a brief, 5-min observation of parent-child interaction (83) and could be utilized in both identification and monitoring the impact of interventions.

Supplementing the common intervention goals, strategies that more precisely meet the needs of the different classes identified should include clinical supports and services for children in the vulnerable group who had delays in early communication skills. Prioritization of strategies that target language promotion and engagement of families in early childhood education and care settings is recommended for the third class where significant social disadvantage was a factor (84). It is important to note that there are broader social and structural inequalities which may make the provision of optimal home-learning environments challenging. Parent support programs, including engagement with health and/or early childhood agencies, need to provide a critical buffer for families where mental health problems are present.

### Strengths and Limitations

ELVS is one of the few large prospective, community cohort studies with a focus on language skills to have collected data from the first year of life, and regularly through the preschool and primary school years. Language skills were measured at multiple time-points using gold standard measures. Using repeated surveys, we measured early life factors pertaining to the child, family, and environment. The robustness of this longitudinal data is rare for complex developmental disorders. These significant strengths have enabled ELVS to make a significant contribution to the literature on language development and disorder.

At the same time, we acknowledge that our sample at 7-years was not reflective of the original ELVS sample or the population more generally, both of which were more disadvantaged. Consequently, the latent classes and the impact of cumulative risk on language outcomes may be different with a more disadvantaged sample. The majority of caregivers in the ELVS sample are mothers and our findings should not be considered representative of father's behaviors or characteristics. Our data reflects the Australian societal characteristics from which it was collected; we accept that some features of which are more comparable to different country contexts than others. ELVS was not designed to assess specific or individual biological or genetic factors, so many of these are not included.

In a large study such as ELVS we did not have the resources to collect parental language input to children, *via* audio or video recordings, at multiple time-points. We acknowledge that this would have provided a rich source of data to further investigate parent-child interaction and is an important direction for future research. Finally, despite our analysis including an extensive set of early life factors, as for any study they are not a complete list of all the possible risk and protective factors may have resulted in different findings to those presented here.

## Conclusion

The cumulative and cluster risk analyses demonstrate the potential to use developmental surveillance to identify children within the first year/s of life who are at increased risk for language difficulties. Next steps will require consultation with practitioners to determine feasibility of this approach. Building a cumulative risk index, our findings demonstrate a “tipping point” when children accumulate four or more risks from a range of early life factors. Many of these risks can be monitored efficiently and repeatedly through existing maternal and child health services. Importantly, parent-child interaction and the home learning environment emerged as common features of clusters of risks. We recommend they be adopted as the common focus for early intervention and universal language promotion programs that target all children and families in a community. Developing policy to implement these recommendations does not require wholesale restructuring of existing services but the careful allocation of resources and training to ensure universal and frequent developmental surveillance is available to all young children and their families regardless of their circumstances.

## Data Availability Statement

The datasets presented in this article are not readily available because data is shared following application to the Chief Investigators of the ELVS study. Requests to access the datasets should be directed to Dr. Penny Levickis, penny.levickis@unimelb.edu.au.

## Ethics Statement

The studies involving human participants were reviewed and approved by Royal Children's Hospital (Melbourne). Written informed consent to participate in this study was provided by the participants' legal guardian/next of kin.

## Author Contributions

PE, CM, and SR conceived and designed the study. PL, EW, and BG analyzed the data. PE, PL, EW, EB, and BG drafted the manuscript. PE, PL, CM, EW, EB, RW, BG, and SR revised the manuscript critically for important intellectual content. All authors contributed to the article and approved the submitted version.

## Funding

The Early Language in Victoria Study was funded by the Australian National Health and Medical Research Council (NHMRC grant numbers 237106, 436958, and 1041947). SR was supported by a Practitioner Fellowship (#1041892). Research at the Murdoch Children's Research Institute was supported by the Victorian Government's Operational Infrastructure Support Program.

## Author Disclaimer

The contents of the published material herein are the sole responsibility of the authors and do not reflect the views of the NHMRC.

## Conflict of Interest

The authors declare that the research was conducted in the absence of any commercial or financial relationships that could be construed as a potential conflict of interest.

## Publisher's Note

All claims expressed in this article are solely those of the authors and do not necessarily represent those of their affiliated organizations, or those of the publisher, the editors and the reviewers. Any product that may be evaluated in this article, or claim that may be made by its manufacturer, is not guaranteed or endorsed by the publisher.
